# Internal quality assessment of blood components at Mansoura university blood transfusion center

**DOI:** 10.1038/s41598-025-17334-1

**Published:** 2025-09-12

**Authors:** Doaa Shahin, Amira M. Tawfeek, Omnia Khaled, Metwaly I. Mortada

**Affiliations:** 1 Haematology Unit, Clinical Pathology Department, Faculty of Medicine, Mansoura University, Mansoura, 35111 Egypt; 2 Clinical Pathology Department, Faculty of Medicine, Damietta University, Damietta, Egypt

**Keywords:** Blood components, Quality control, Red blood cell concentrates, Fresh frozen plasma, Platelet concentrates, Cell biology, Health care, Medical research

## Abstract

In modern blood banking, quality control of blood products ensures the timely availability of a blood component of high-quality yield with maximum efficacy and minimal risk to potential recipients. A prospective cross-sectional study was carried out at Mansoura University Hospital Blood Bank (MUHBB) aiming to assess the internal quality control (IQC) of 300 units of each blood components, red blood cell concentrates (RBCs), fresh frozen plasma (FFP), and platelet concentrates (PCs), and to explore their compliance with The Egyptian National Blood Transfusion Services (NBTS ),The American Association of Blood Banks (AABB), and Council of Europe (CE) standard criteria. IQC of different blood products was accepted if > 90% of units were fulfilled the standard requirements. The results of our study show that the total compliance of RBCs was 93.3% according to NBTS and CE standards and 96% as regards AABB standards. The total compliance of FFP was 94.0% according to NBTS and CE standards and 96.0% as regards AABB standards. While the total compliance of PCs was 96.0% according to NBTS and AABB standards and 93.3% as regards CE standards. Continuous improvement is recommended to decrease the percentage of noncompliance in the QC parameters included in our study and to uplift the blood transfusion practices.

## Introduction

Blood transfusion service is an essential component of healthcare in the context of treating disease and restoring health. It relies on human blood as a fundamental resource for humanitarian purposes and not for commercial purposes. The government is tasked with establishing different regulations to guarantee the availability of blood or blood components that are safe, easily accessible, and affordable by the community^1^.

IQC plays a crucial role in quality assurance (QA) in all laboratory services. It is the predetermined set of procedures that are carried out continuously for evaluation of routine work, so as to assess the performance standards. Ensuring QC standards in blood banks plays an important role in reducing occurrence of adverse blood reactions. Consistent periodic quality assessment of blood components is aimed to track variations in manufacturing processes, product quality and ensure that manufacturing steps meet established criteria for approval^2,3^. In modern blood banking, quality control measures for blood products guarantee the timely availability of efficiently processed blood component with enhanced effectiveness and reduced transfusion-related complications. This goal can be achieved by ensuring the proper and strict implementation of the procedures that focused on producing safe and efficient blood components^4^. There are many organizations which set QC standards for blood components such as Egyptian National Blood Transfusion Services (NBTS), The American Association of Blood Banks (AABB), and Council of Europe (CE).

Therefore, this study aimed to assess the internal quality control (IQC) of different blood products (red blood cell concentrates, platelets, fresh frozen plasma) processed in Mansoura University hospital blood bank and to explore their compliance with The Egyptian NBTS, AABB, and CE standard criteria.

## Patients and methods

A prospective crosssectional study was carried out at the Mansoura University Hospital Blood Bank., from December 2022 to December 2023. During this period 30,000 units of whole blood were collected from healthy voluntary non-remunerated blood donors and non-regular (family/replacement) donors in sterile triple blood bags containing 63 ml CPDA-1 (citrate phosphate dextrose adenine-1) as anticoagulant, and the satellite bag for platelets is approved for five-day platelet storage. All donors provided signed informed consent prior to donation. Whole blood units were processed within 6 to 8 h post-collection using a two-step centrifugation technique (an initial light spin followed by a heavy spin) in a refrigerated blood component centrifuge to separate the primary components: red blood cell concentrates (RBCs), platelet concentrates (PCs), and fresh frozen plasma (FFP). Platelet concentrates were prepared using the platelet-rich plasma (PRP) method, which involves an initial low-speed centrifugation to separate the PRP into a satellite bag for platelets, while the PRBCs remain in the primary collection bag and stored at 1–6 °C. This is followed by a high-speed centrifugation of the PRP to sediment the platelet pellet, after which the supernatant plasma is transferred to an attached empty bag and frozen within 8 h and stored at − 30 °C. The platelet concentrate is stored at RT (20–24 °C) with gentle agitation in platelet storage incubator.

Of the total collected units, 300 units (1% of the total) of each blood component (RBCs, PCs and FFP) were randomly selected throughout the study period. IQC testing was performed once weekly for each product, and all components were evaluated near the expiry date.

All blood units (RBCs, PCs and FFP) from donors in Mansoura University hospital blood bank were included in the study with exclusion of blood units which were not completed during the process of donation, leucocyte depleted blood products, Washed blood products, and single donor apheresis platelet (SDAP).

**RBCs** units were assessed for volume, hemoglobin, hematocrit (HCT) and the presence of hemolysis; evaluation of **FFP** units included unit volume, Factor VIII concentration, and physical integrity (evidence of leakage and visual changes); and **PCs** units were evaluated for volume, pH, platelet yield, RBCs contamination, Residual leukocytes, Swirling phenomenon and culture. Quality control parameters of RBCs, PCs and FFP were compared with the Egyptian NBTS, AABB and CE standard requirements for their compliance.

*Quality control requirements according to the Egyptian NBTS 4 Th edition*,* 2023: RBCs*: Volume of RBCs unit should be 280 ml plus or minus 50 ml, hematocrit should be 60–75% and also no visual sign of hemolysis should be detected. *FFP*: Volume should be > 170 ml, factor VIII should be > 0.7 IU/ml and no abnormal color should be seen in FFP unit. *PCs*: Volume should be 50–60 ml. Platelet count per unit should be Minimum 5 × 10^10^ and pH at the end of the recommended shelf life should be 6.4–7.4 in at least 90% of units sampled^5^.

*Quality control requirements according to AABB Technical Manual 21 st edition*,* 2023: RBCs*: QC of RBCs units includes that hemoglobin content and hematocrit per RBC unit vary because of differences between donors and between blood component manufacturing methods. Total hemoglobin content is not directly regulated but has a lower limit of 45 g per unit in the United States. Hematocrit in RBCs from whole blood in anticoagulant-preservative citrate-phosphate –dextrose (CPD)or citrate-phosphate –dextrose-dextrose (CP2D) is 65–85% and in CPDA-1 is < 80%. The use of additive solutions reduces the hematocrit to approximately 55–65%. Also, no visual sign of hemolysis should be detected. *FFP*: Volume should be 200–250 ml. FFP is generally not used in developed countries but developing countries largely depend on FFPs for various inherited coagulation defects. In view of its limited use, AABB do not recommend any standards for its testing^6^. *PCs*: Volume should be 50–70 ml, platelet count per unit should be > 0.6 × 10^11^, pH at the end of shelf life should be > 6.4, swirling present and residual leucocytes per unit should be ≤ 0.2 × 10^9^. All these requirements should be present in a minimum of 90% of units tested^7^.

*Quality control requirements according to CE 21 st edition*,* 2023: RBCs*: Volume of RBCs unit should be 280 ml plus or minus 50 ml, hematocrit should be 65–75%, hemoglobin should be ≥ 45 gm per unit and haemolysis at the end of storage should be < 8% of red cell mass. *FFP*: Volume should be stated volume ± 10% (Units measured and found to be 340 mL should only be issued for transfusion under concessionary release), factor VIII should be > 0.7 IU/ml, Residual cellular content must meet specific limits: red cells should be less than 6.0 × 10⁹/L, leukocytes less than 0.1 × 10⁹/L, and platelets less than 50 × 10⁹/L. Furthermore, FFP units must be free from any leakage or visible abnormalities. *PCs*: Volume should be 50–70 ml, platelet count per unit should be > 0.6 × 10^11^, pH at the end of shelf life should be > 6.4, swirling present and residual leucocytes per unit should be ≤ 0.2 × 10^9^. All these requirements should be present in a minimum of 90% of units tested^8^.

### Statistical analysis

Data was entered and analyzed using IBM-SPSS software (IBM Corp. Released 2017. IBM SPSS Statistics for Windows, Version 25.0. Armonk, NY: IBM Corp.). Qualitative data was expressed as N and percentage (%). Quantitative data was initially tested for normality using Kolmogorov-Smirnov test. Quantitative data was expressed as mean ± standard deviation (SD) if normally distributed or median and range if not. All data was classified to fulfilling or not fulfilling according to agreement with different standard requirements. Compliance was calculated as units fulfilling all parameters with standard requirement divided by whole units’ number. Accepted if > 90% of units were fulfilled the standard requirements.

## Results

A total of 300 whole blood derived RBCs were tested for QC and revealed that (Table 1); as regards the NBTS requirements, the volume of 284 bags out of 300 (94.7%) was in conformance with NBTS standards, also hematocrit of 292 bags (97.3%) was harmonious with NBTS standards. Regarding AABB standard requirements, hematocrit of 298 bags (99.3%) was consonant with AABB standards. Regarding CE standard requirements, the volume of 284 bags out of 300 (94.7%) was in conformance with CE standards, hematocrit of 288 bags (96.0%) was consonant with CE standards. Hemoglobin of 290 bags (96.7%) was in concordance with AABB and CE standards except for 10 bags (3.3%) that all of them had hemoglobin below the desired range. No visual signs of hemolysis were detected in 299 bags (99.7%), which is consistent with NBTS standards, except for one bag (0.3%) that showed visible hemolysis. However, visual assessment is not considered a standard method in the AABB guidelines. The CE Guide recommends that hemolysis be evaluated at the end of storage and expressed as a percentage of red cell mass.

**Table 1:** Comparison of quality control result of red blood cell concentrates prepared from whole blood units (*n* = 300) according to the Egyptian NBTS, AABB and CE standard requirements.

Comparison of QC results of RBCs from whole blood units according to the NBTS, AABB and CE standard requirements (*n* = 300)					
Parameters (per bag)		Volume (ml)	Hematocrit (%)	Hemoglobin (gm)	Hemolysis
**The present study**	**Mean ± SD** **range**	258.8 ± 15.99	66.4% ± 3.24	57.2 ± 3.94	Detected in only 1 unit
		(200–296)	(45.0%−82.0%)	(40.4–64.2)	
**Units fulfilling NBTS criteria**		284 (94.7%)	292 (97.3%)	NA	299 (99.7%)
**Units not fulfilling NBTS criteria**		16 (5.3%)	8 (2.7%)	NA	1 (0.3%)
**Units fulfilling AABB criteria**		NA	298 (99.3%)	290 (96.7%)	NA
**Units not fulfilling AABB criteria**		NA	2 (0.7%)	10 (3.3%)	NA
**Units fulfilling CE criteria**		284 (94.7%)	288 (96.0%)	290 (96.7%)	NA
**Units not fulfilling CE criteria**		16 (5.3%)	12 (4.0%)	10 (3.3%)	NA

NA = data not available.

As regard the QC of 300 whole blood derived FFP (Table 2); the volume of 290 bags out of 300 (96.7%) was in conformance with NBTS standards. Regarding AABB standards and CE standard requirements, the volume of 288 bags (96.0%) was consonant with both standards. Factor VIII of 282 bags (94.0%) was harmonious with NBTS and CE standards. No visual change or leakage in all units tested.

Regarding 300 whole blood derived *PCs*: QC results (Table 3); the volume and pH of 300 bags, were in concordance with the NBTS, AABB standards and CE requirements. The platelet count per units of 288 bags out of 300 (96.0%) was in conformance with NBTS and AABB standards except for 12 bags (4.0%) that all had platelet count below desired range. As regard CE standard requirements the platelet count per unit of 280 bags out of 300 (93.3%) was in conformance with CE standards. Also, no visible RBCs and negative culture in all units tested which both were in conformance with AABB standards. Also, residual WBCs per unit were below the accepted range in all units tested, and the swirling phenomenon was present in 100.0% of units tested which both were in conformance with CE standards.

The results of our study show that the total compliance of RBCs was 93.3% according to NBTS and CE standards and 96% as regards AABB standards. A total of 282 FFP bags (94.0%) out of 300 were in compliance with NBTS and CE standard requirements while 288 FFP bags (96.0%) were in compliance with AABB standard requirements. The total compliance of PCs was 96.0% according to NBTS and AABB standards and 93.3% as regard CE standards. (Table 4).

**Table 2:** Quality control parameters of fresh frozen plasma prepared from whole blood units (*n* = 300) according to the Egyptian NBTS, AABB and CE standard requirements.

Comparison of the result of QC of FFP from whole blood units according to the NBTS, AABB and CE standard requirements (*n* = 300)					
Parameters (per bag)		Volume (ml)	Factor VIII (IU/ml)	Visual change	Leakage
**The present study**	**Mean ± SD** **range**	206.7 ± 14.44	0.913 ± 0.211	No abnormal color or visible clots in 100% of units tested.	No leakage in any part of container in 100% of units tested.
		(150–250)	(0.39-2.00)		
**Units fulfilling NBTS criteria**		290 (96.7%)	282 (94.0%)	300 (100.0%)	NA
**Units not fulfilling NBTS criteria**		10 (3.3%)	18 (6.0%)	0 (0.0%)	NA
**Units fulfilling AABB criteria**		288 (96.0%)	NA	NA	NA
**Units not fulfilling AABB criteria**		12 (4.0%)	NA	NA	NA
**Units fulfilling CE criteria**		288 (96.0%)	282 (94.0%)	300 (100.0%)	300 (100.0%)
**Units not fulfilling CE criteria**		12 (4.0%)	18 (6.0%)	0 (0.0%)	0 (0.0%)

NA = data not available.

**Table 3:** Comparison of quality control result of platelet concentrates prepared from whole blood units (*n* = 300) according to the Egyptian NBTS, AABB and CE standard requirements.

Comparison of the result of QC of PCs from whole blood units according to the NBTS, AABB and CE standard requirements (*n* = 300)								
Parameters (per bag)		Volume (ml)	Platelet per units	pH at the end of shelf life	Residual WBCS per unit	Appearance	Culture	Swirling
**The present study**	**Mean ± SD** **range**	54.4 ± 3.45	7.35 × 10^10^ ± 1.	6.6 ± 0.24	0.015 × 10^9^± 0.007	No visible RBCS in 100% of units	Negative in 100% of units	Present in 100% of units
		(50–60)	(4 × 10^10^ −10 × 10^10^)	(6.5-7.0)	(0.005 × 10^9^−0.031 × 10^9^)			
**Units fulfilling NBTS criteria**		300 (100.0%)	288 (96.0%)	300 (100.0%)	NA	NA	NA	NA
**Units not fulfilling NBTS criteria**		0 (0.0%)	12 (4.0%)	0 (0.0%)	NA	NA	NA	NA
**Units fulfilling AABB criteria**		300 (100.0%)	288 (96.0%)	300 (100.0%)	NA	300 (100.0%)	300 (100.0%)	NA
**Units not fulfilling AABB criteria**		0 (0.0%)	12 (4.0%)	0 (0.0%)	NA	0 (0.0%)	0 (0.0%)	NA
**Units fulfilling CE criteria**		300 (100.0%)	280 (93.3%)	300 (100.0%)	300 (100.0%)	NA	NA	300 (100.0%)
**Units not fulfilling CE criteria**		0 (0.0%)	20 (6.7%)	0 (0.0%)	0 (0.0%)	NA	NA	0 (0.0%)

NA = data not available.

**Table 4:** Total compliance of RBC, FFP and PCs units according to the Egyptian NBTS, AABB and CE standard requirements.

Total compliance of RBC, FFP and PCs units according to the Egyptian NBTS, AABB and CE standard requirements			
Parameters (per bag)	RBC	FFP	PCs
**Egyptian NBTS requirements (4th edition 2023)**	280 (93.3%)	282 (94.0%)	288 (96.0%)
**AABB standards requirements (21st edition 2023)**	288 (96.0%)	288 (96.0%)	288 (96.0%)
**CE standard requirements (21st edition 2023)**	280 (93.3%)	282 (94.0%)	280 (93.3%)

## Discussion

An effective quality system should be developed by every blood center to guarantee the implementation of quality strategies. The quality procedure ought to cover all aspects of its activities and confirm traceability, from recruiting and selecting all blood donors to blood components and the transfusion of blood to recipients. It also needs to reflect the structure, desires and abilities of the blood center, as well as the hospitals’ needs and patients that they serve. This should provide an outline within which blood transfusion service activities are determined and executed in a high-quality focused state and constantly observed to improve results^9^.

The quality control of blood products is the primary method to evaluate product viability, and subsequently, it can modify production methods when needed. The present study was designed to assess the IQC of blood products prepared in our blood bank and to compare the findings with established national and international standards. To the extent of our knowledge, this study was the first to be reported by our nation.

The QC results of volume of RBCs units, 284 bags out of 300 (94.7%) were in conformance with NBTS and CE standards with the exception of 16 bags (5.3%) that all had volume below 230 ml. Low volume units in our study might be due to technical error during separation of our blood components so, the technicians were advised to check the volume correctly according to the QC standards during separation of blood components. Low volume of whole blood units was excluded because in our protocol we had double checked the volume of whole blood units before separation of our blood components. Regarding the QC results of hematocrit 292(97.3%), 298 bags (99.3%) and 288 bags (96.0%) out of 300 were consonant with NBTS, AABB and CE standards respectively. NBTS, 8 bags (2.7%) were out that 6 of them had hematocrit below the desired range and the rest two had slightly higher hematocrit above desired range. Regarding AABB standards requirements, 2 bags (0.7%) were out that had slightly higher hematocrit above the desired range. Regarding CE standard requirements, 12 bags (4.0%) were out that 10 of them had hematocrit below the desired range and the rest two had slightly higher hematocrit above desired range. The problem with hematocrit in our study might be due to improper plasma volume left at the end of 1 st rotation during separation. Patel et al. observed and recommended that in RBC, the problem with hematocrit content, so the technicians were advised to make sure that at least 50 ml plasma left at the end of 1 st rotation during separation. So that RBCs do not get too much concentrated or diluted^10^.

The QC results of hemoglobin 290/300 bags (96.7%) were in concordance with AABB and CE standards except for 10 bags (3.3%) that all of them had hemoglobin below the desired range. Low hemoglobin levels were due to might be attributed to lower normal hemoglobin levels in our donor population. No visual sign of hemolysis was detected in 299 units (99.7%) which was in conformance with NBTS requirements with the exception of 1 bag (0.3%). This might be due to delay between collection and separation, rapid centrifugation (including mixing of anticoagulant with blood), large variation in centrifugation speeds, rapid resuspension of PRBCs in additive solutions, and variations in blood storage bag configurations or compositions.

A total of 300 whole blood derived FFP were tested for QC and revealed that; as regard The NBTS requirements, the volume of 290/300 bags (96.7%) was in conformance with NBTS standards with the exception of 10 bags (3.3%) that all had volume below 170 ml. Regarding AABB standards and CE standard requirements, volume of 288 bags (96.0%) was consonant with both standards except for 12 bags (4.0%) that all of them had volume below the desired range. Low volume units in our study might be due to technical errors during separation of our blood components so, the technicians were advised to check the volume correctly according to the QC standards during separation of blood components. Concerning the QC results of factor VIII, factor VIII of 282 bags (94.0%) was harmonious with NBTS and CE standards with the exception of 18 bags (6.0%) that all of them had factor VIII below the desired range. Low factor VIII levels in 18 bags (6.0%) of our FFP units might be due to slow freezing process in 12 bags (4%) of our FFP units.

Dhantole et al. reported that in slow freezing conditions, with freezing duration longer than one hour, ice tends to form at the edges, while solutes become increasingly concentrated in the center. So, factor VIII exposed to high salts concentration for a long period, causing inactivation of it. On the other hand, rapidly reducing the temperature to − 30 °C or below within one hour helps preserve Factor VIII activity by allowing ice formation to outpace solute migration. This entraps solute clusters uniformly within the ice, minimizing their interaction with Factor VIII^11^. As regard the other 6 bags (2%) of our FFP with low factor VIII levels, longer donation time (exceeding 15 min) was the cause. The CE guidelines reported that if whole blood collection time more than 15 min, the plasma should not be used for direct transfusion or for the coagulation factors preparation^8^. No visual change was detected in all units tested which was in conformance with NBTS and CE standards. Also, no leakage was detected in all units tested which was in conformance with CE standards.

A total of 300 whole blood derived PCs were tested for QC and revealed that the volume and pH 100% of bags were in concordance with the NBTS, AABB standards and CE requirements. As regard the platelet count per unit, 288/300 bags (96.0%) were in conformance with NBTS and AABB standards except for 12 bags (4.0%) that all had platelet count below desired range. As regard CE standard requirements, the platelet count per units of 280 bags out of 300 (93.3%) was in conformance with CE standards except for 20 bags (6.7%) that all had platelet count below desired range. Low platelet yield was due to longer donation time (exceeding 15 min) in 6 bags (2%) of our PCs units.

The CE guidelines reported that if the duration of the bleeding for whole blood collection is longer than 15 min, the blood should not be used for the preparation of platelets. The other cause of low platelet yield units might be attributed to lower normal platelet count in our donor population. While the QC results of residual WBCs per unit were 0.015 × 10^9^ ± 0.007 below the accepted range (≤ 0.2 × 10^9^) in 100.0% of units tested which were in conformance with CE standards. The swirling phenomenon was present in 100.0% of units tested which was in conformance with CE standards. Also, no visible RBCs was detected in 100.0% of units tested which was in conformance with AABB standards. Regarding the QC results of culture, culture was negative in 100.0% of units tested which was in conformance with AABB standards^5,7^.

The results of our study show that the total compliance of RBC was 93.3% according to NBTS and CE standards and 96% as regard AABB standards. The total compliance of FFP was 94.0% according to NBTS and CE standards and 96.0% as regard AABB standards. The total compliance of PCs was 96.0% according to NBTS and AABB standards and 93.3% as regard CE standards.

These findings are consistent with prior research (Sultan et al., 2018) who evaluated 100 units (a total of 400 units; RBCs, PCs, FFP and CP) and revealed that evaluated HCT of PRBCs met the AABB standard in 98.0% of units tested. Regarding RDPS, this research assessed PH and platelet yield, which noted that mean pH was 7.5 ± 0.08 (met criteria in 98.0% of units) and mean platelet yield was 8.87 ± 3.40 × 10^9^/L (met criteria in 94.0% of units). Culture was negative in 97.0% of units tested, but after subsequent repetition was negative which excludes any bacterial contamination of units. The author explained this variation by faulty sampling procedure. Mean factor VIII and fibrinogen levels were (84.24 ± 15.01 and 247.17 ± 49.69) for FFP and (178.75 ± 86.30 and 420.7 ± 75.32) for CP respectively, which were in conformity with AABB standard in 100% of units concerning fibrinogen level in both FFP and CP and 95% for FFP and 96% for CP concerning factor VIII level [6].

These go in harmony with H. Patel et al. who performed monthly quality control of total 1302 units (473 RBCs assessed for HCT, 255 PCs assessed for yield, 484 FFP and 90 cryoprecipitate evaluated for volume, factor VIII and fibrinogen levels) in the period between December 2019 to November 2021 according to National accreditation Board for Hospital and Healthcare^12^. He demonstrated that in case of RBCs, PCs and culture criteria matched the standard in 95.0%, 95.0% and 100.0% respectively. In case fibrinogen level 100% of FFP and CP met the standard, while factor VIII level 99.2% of FFP and 91.2% of CP could meet the standard^13^.

Another study published by K. Patel assessed volume (365.1 ± 53.05 ml), HCT (62.09 ± 6.56%), Hb (19.22 ± 2.66 g/dl) and RBCs count (7.20 ± 1.09 × 10^12^ for RBCs, yield (7.47 ± 1.95 × 10^9^), volume (62.94 ± 5.75 ml), culture (sterile in 100.0%) and swirling (present in 100.0%) for RDPs, fibrinogen and factor VIII level for FFP (271.23 ± 89.32 gm/L and 83.98 ± 16.11 IU/unit respectively) and CP (280.6 ± 69.83 gm/L and 81.83 ± 17.23 IU/unit respectively) with regard to AABB standard and achieved matching with criteria^10^.

Study from different centers in three countries in Latin America by Cardona et al. who analyzed 1402 RBC (for volume, HCT, leucocytes and Hb) and 1245 platelet concentrates (for volume, platelet count, pH and leucocytes) obtained by buffy coat method conforming with European Directorate for the quality of Medicine and Healthcare (EDQM)^14^ and reported that total compliance for RBC criteria for three centers (91.7%), and for platelet concentrates for three centers (94.0%)^15^.

This was in agreement with Coêlho et al. assessed aggregation and other quality control markers (volume, count, pH, swirling, leucocyte count and microbiological examination) as regards their technical standards, swirling was detected in all platelet units (*n* = 80), while only 4 units had erythrochromia. The mean volume was around 60 ml. There was an increase in platelet count on the third day compared to the first day which explained by platelet fragmentation. Also, a similar increase in pH was observed from 7.4 to 7.7. All platelet units were sterile. Leucocyte count was in normal range which is very important as leukocytes compete for available oxygen^16^.

In contrast to these results previous two other studies (Huamani-Chacchi et al. and Fasola et al.) had Huamani-Chacchi et al. evaluated 133 platelets unit as regards the National Program of Hemotherapy and Blood Banks standards. As regards platelet count, 75 units were below 5.5 × 10^10^ which not met the compliance. On the other hand, the other four parameters (swirling effect, pH, volume and leukocyte count) met the criteria^17^. Fifty platelet concentrates were assessed for (pH, swirling, volume, count and WBCs count) at the University College Hospital (U.C.H.) Ibadan, Nigeria by Fasola et al. and documented that swirling was detected in 72.0% of units, average volume was 18.52 ml/bag. Only 35% of units had count > 5.5 × 10^10^/L. The author indicated that the need to improve quality of platelet units for therapeutic level^18^.

### At the end of the discussion section

- Our findings underscore the need for Continuous Quality Improvement (CQI) initiatives to reduce noncompliance in blood component quality. CQI should encompass all stages of blood collection and processing, with clearly defined quality indicators that are consistently monitored, documented, and evaluated. Key additions to our QC protocol should include the assessment of hemolysis in RBCs at the end of storage (expressed as a percentage of red cell mass), the evaluation of residual cells in plasma as recommended by the EDQM guidelines, and routine monthly comparisons of QC results to detect trends or deficiencies. Prompt corrective actions must be taken when issues are identified. Regular staff training and internal audits conducted by qualified, independent personnel are essential. The use of Statistical Process Control (SPC), including control charts, is strongly recommended to enable proactive, data-driven decision-making. Although full implementation is currently constrained by limitations in infrastructure and staff training, these measures are critical for improving compliance and enhancing the safety and quality of blood transfusion services.

## Conclusion

From our results, it can be concluded that the blood components’ quality had prepared at Mansoura University Hospital Blood Bank meet the national and international standards. It is also essential to highlight the importance of QC programs in preparing blood components throughout blood transfusion centers, which will favor the adequate standardization of all countries’ implementation processes.

The present study recommends that:

Quality assessment be established as a standard protocol across all blood transfusion centers. Comprehensive quality analysis records should be consistently maintained, and a broader set of quality indicator parameters should be incorporated to ensure high standards in blood transfusion practices. Furthermore, continuous quality improvement initiatives are essential to reduce the rate of noncompliance observed in the quality control parameters assessed in this study, thereby enhancing overall transfusion practices.

## Data Availability

The datasets used and/or analysed during the current study available from the corresponding author on reasonable request.
